# Assessing and Validating an Educational Resource Package for Health Professionals to Improve Smoking Cessation Care in Aboriginal and Torres Strait Islander Pregnant Women

**DOI:** 10.3390/ijerph14101148

**Published:** 2017-09-29

**Authors:** Yael Bar-Zeev, Michelle Bovill, Billie Bonevski, Maree Gruppetta, Jennifer Reath, The ICAN QUIT in Pregnancy Pilot Group, Gillian S. Gould

**Affiliations:** 1 School of Medicine and Public Health, University of Newcastle, Newcastle, NSW 2300, Australia; michelle.bovill@newcastle.edu.au (M.B.); billie.bonevski@newcastle.edu.au (B.B.); maree.gruppetta@newcastle.edu.au (M.G.); gillian.gould@newcastle.edu.au (G.S.G.); 2 Department of General Practice, Western Sydney University, Campbelltown, NSW 2560, Australia; J.Reath@westernsydney.edu.au; 3 The ICAN QUIT in Pregnancy Pilot Group; icanquitinpregnancy@gmail.com

**Keywords:** smoking cessation, indigenous health, health professionals, pregnancy

## Abstract

Australian Aboriginal pregnant women have a high smoking prevalence (45%). Health professionals lack adequate educational resources to manage smoking. Resources need to be tailored to ensure saliency, cultural-sensitivity and account for diversity of Indigenous populations. As part of an intervention to improve health professionals’ smoking cessation care in Aboriginal pregnant women, a resource package was developed collaboratively with two Aboriginal Medical Services. The purpose of this study was to assess and validate this resource package. A multi-centred community-based participatory 4-step process (with three Aboriginal Medical Services from three Australian states), included: (1) Scientific review by an expert panel (2) ‘Suitability of Materials’ scoring by two Aboriginal Health Workers (3) Readability scores (4) Focus groups with health professionals. Content was analysed using six pre-determined themes (attraction, comprehension, self-efficacy, graphics and layout, cultural acceptability, and persuasion), with further inductive analysis for emerging themes. Suitability of Material scoring was adequate or superior. Average readability was grade 6.4 for patient resources (range 5.1–7.2), and 9.8 for health provider resources (range 8.5–10.6). Emergent themes included ‘Getting the message right’; ‘Engaging with family’; ‘Needing visual aids’; and ‘Requiring practicality under a tight timeframe’. Results were presented back to a Stakeholder and Consumer Aboriginal Advisory Panel and resources were adjusted accordingly. This process ensured materials used for the intervention were culturally responsive, evidence-based and useful. This novel formative evaluation protocol could be adapted for other Indigenous and culturally diverse interventions. The added value of this time-consuming and costly process is yet to be justified in research, and might impact the potential adaption by other projects.

## 1. Introduction

Aboriginal and Torres Strait Islander pregnant women (hereafter referred to “Aboriginal” women with acknowledgement of the distinct cultures) have the highest smoking rate during pregnancy in Australia (45%) [1], and are three times more likely to smoke during pregnancy compared to non-Aboriginal pregnant women [2]. Smoking during pregnancy is the most important preventable risk factor for poor maternal and infant health outcomes, including miscarriage, growth restriction, stillbirth and pre-term birth [3].

Lack of support from health professionals is a common barrier to smoking cessation in different vulnerable groups, including the Aboriginal population [4]. Aboriginal women report that they receive inconsistent messages from health professionals during pregnancy [5]. Health professionals also report many challenges to providing smoking cessation care in pregnancy [6,7], including insufficient topic knowledge, low confidence in counselling, shortage of time, and little optimism about the effectiveness of interventions. In a recent national cross-sectional survey of Australian General Practitioners (GPs) and Obstetricians, insufficient resources were reported as one of the main barriers to smoking cessation care in pregnant women [8]. A unique barrier in pregnancy is the lack of a strong evidence base on the safety and efficacy of nicotine replacement therapy (NRT), which might impact clinicians’ confidence and skills to prescribe NRT [9]. These challenges were reported from studies conducted among the general population, and are not specific to the Aboriginal population.

Printed self-help materials have been shown to improve smoking cessation rates (RR 1.19, 95% CI 1.04–1.37) [10]. Similarly, printed educational materials intended for health professionals can also have a positive impact on their practice (median absolute risk difference in practice outcomes 0.02, range 0–0.11) [11]. When developing educational resources, many considerations need to be taken into account to ensure resources are actually useful and effective, including readability level, appearance and organization of the data [12].

### 1.1. Tailoring Educational Resources

Tailoring messages for a specific target population might improve their usefulness and effectiveness [13]. Previous systematic reviews exploring health promotion interventions that were adapted for ethnic minority populations have concluded that currently there is a lack of evidence for effectiveness of tailoring [14,15]. However, both reviews agree that adapting interventions might increase salience, acceptability and uptake. Furthermore, none of these included studies with Indigenous populations. Research reveals that although generic (intended for the general population) messages impact Indigenous populations, there is a preference for culturally targeted messages [16]. Formative research ensures the development of targeted, culturally appropriate, health messages that work [17,18]. In the past few years, research done specifically with Aboriginal pregnant women has shed light on some of the myths and beliefs about smoking during pregnancy that are a barrier to quitting [19,20,21,22]. Additionally, in developing a suitable intervention, the challenge of designing appropriate anti-tobacco messages that account for the diversity of Aboriginal People has been outlined [19]. Conducting a pre-test of messages is associated with increased rigour in developing programs targeted to an Aboriginal population [23]. Daley et al. [24] describe in detail a rigorous assessment process of educational material they developed for a smoking cessation intervention for American Indians. These educational materials were then used as part of a randomized controlled study showing promising results in increasing smoking cessation rates [25].

This study comprised the first phase of the Indigenous Counselling and Nicotine (ICAN) Quit in Pregnancy trial [26]. The ICAN QUIT in Pregnancy intervention aimed to improve health professionals smoking cessation care with Aboriginal pregnant women who smoke and included three one-hour webinar training sessions for health professionals, an educational resource package, and free oral NRT [26]. Phase 1 of the ICAN QUIT in Pregnancy trial focuses on the development and pre-testing of the educational resources.

### 1.2. Aims

To assess the accuracy, readability, cultural acceptability and perceived usability of a collaboratively developed educational resource package to aid health professionals’ smoking cessation care in pregnant Aboriginal women.

## 2. Materials and Methods

### 2.1. The Indigenous Counselling and Nicotine (ICAN) Quit in Pregnancy Trial

This intervention is based on the previously published ABCD guidelines (Ask about smoking; Brief advice to quit; Cessation support; Discuss the psychosocial context of smoking) with an expedited offer of NRT [27]. The authors worked collaboratively with two Aboriginal Medical Services [28] to develop this intervention. A Stakeholder and Consumer Aboriginal Advisory Panel (SCAAP) and a smaller Working Party (Aboriginal and non-Aboriginal staff from the two medical services, and Aboriginal female community members) guided the development of the educational resource package [28]. A whole-of-service approach was intended, to train all of the health professionals including GPs, midwives, Aboriginal Health Workers (AHW), and other allied health professionals. Thus, the educational resource package [29] needed to suit health professionals with different educational needs.

A main focus of the intervention was to address clinician’s low confidence and skills to prescribe NRT [9]. The latest 2015 Cochrane review focusing on pharmacological interventions for smoking cessation during pregnancy found that NRT improved cessation rate by 40% (Relative Risk (RR) 1.43, 95% CI 1.03–1.93). However, when restricting the meta-analysis to only placebo controlled studies, a lower, not significant cessation rate of 28% (RR 1.28, 95% CI 0.99–1.66) was found [30]. Nicotine has been implicated in animal studies to affect foetal development; however, human studies have not found any harmful effects [9,30]. Therefore, experts and clinical guidelines recommend the use of NRT for pregnant women who smoke and have been unsuccessful quitting without medication [9].

*Design:* A multi-centre community based participatory research project.

*Sample:* Three participating sites, from three different states in Australia–South Australia (SA); New South Wales (NSW); and Queensland (Qld). All sites were Aboriginal Community Controlled Health Services (ACCHS), dedicated to healthcare delivery to Aboriginal communities, and overseen by an Aboriginal Community Board of Directors [31].

*Materials to be assessed:* The educational resource package [29] included resources intended for the health professionals, the pregnant women (patients), and both (Box 1).

Box 1 **The educational resource package.**

**1. For the Health Professionals**(a)A detailed treatment manual covering the ABCD approach [27], including specific behaviour change techniques recommended for use to support pregnant women to quit smoking [32]; and detail practical guidelines on the use of Nicotine Replacement Therapy (NRT) in pregnancy.(b)Desktop guide –to be used as a prompt to perform the ABCD, and included an NRT treatment algorithm.**2. For the Pregnant Women (Patients)**Brochures on three specific topics—‘Quitting in Pregnancy’, ‘Triggers’, and ‘Smoke Free Homes’ and also five information sheets on the different NRT products (Patches, Gum, Lozenge, Inhalator, and Oral Spray). To increase engagement and understanding in a population that may have low literacy skills [33], the brochures include short videos embedded into them that could be downloaded using a free App. Topics covered by these videos included: *‘how smoke affects the baby when pregnant’, ‘myths of smoking when pregnant’, ‘explaining smoking triggers and how to address these’* and *‘how to use the different NRT products’.***3. For Both the Health Professionals and the Pregnant Women**A flipchart to be used by the health professional during the consultation with the pregnant woman. A visual side for the women with minimal text, and the reverse side for the health professional as a more detailed prompt on the topics to cover during the consultation. To increase engagement, the visual side for the women included photographs of Aboriginal women from a range of communities in Northern Territory, Victoria and New South Wales.

### 2.2. Procedures

The resources were assessed by a four step evaluation process, based on Daley et al. [24].

#### 2.2.1. An Expert Scientific Panel

Eleven experts were invited to participate, with ten agreeing to review the resources. Feedback was provided by eight of these, from different areas of expertise (Tobacco Treatment Specialist specializing in maternity care; Tobacco Treatment Specialist experienced with providing training to physicians and allied health professionals in the area of smoking cessation; a member of the Royal Australia and New Zealand College of Obstetrics and Gynaecology—Indigenous Women’s Group; An experienced international researcher in randomized controlled trials with NRT and pregnant women; a member of the Congress of Aboriginal and Torres Strait Islander Nurses and Midwives; a Torres Strait Islander General Practitioner; an appointed representative of the Aboriginal Health and Medical Research Council).

Invited experts received a digital and hardcopy of all the education resources. They were not provided with any structured feedback form, but rather asked via email to review the material and provide comments. Experts were instructed to provide the feedback in any way that they found acceptable–direct comments on the copies provided and/or separately in a word document or email. Any changes and/or comments that were made by the experts, for each separate resource, were coded by one researcher (YBZ) into one of six pre-determined themes—Attraction, Comprehension, Self-Efficacy, Graphics and Layout, Cultural acceptability, and Persuasion [34]. These themes have been previously identified as important when assessing health education material to be used specifically with populations with low literacy [34]. Thereafter, for each theme, a summary of the main recommendations was generated and distributed to all other researchers for feedback.

#### 2.2.2. The Suitability of Materials (SAM) Assessment Method Score

The SAM score is a validated systematic process to objectively evaluate the suitability of health education material [34]. It includes 22 items covering 6 themes (Content; Literacy demand; Graphics; Layout and topography; Learning, stimulation, and motivation; Cultural appropriateness). For each item, a score between 0 (not suitable) to 2 (superior) is given. The total score is then calculated (0–39% not suitable material; 40–69% adequate; 70–100% superior).

The SAM was performed by 1–2 staff members from each participating site on a sample of the patient brochures. In total, four staff members participated—three AHW, and one non-Aboriginal Tobacco action worker. The service selected the staff member to perform the SAM rating. For each brochure, two separate SAM ratings (each from a different site) was performed. Mean scores for each brochure and an overall inter-rater agreement score (Kappa) were calculated.

#### 2.2.3. Readability Testing

The text from all of the educational materials was entered into an online tool (Readable.io). Since the visual side of the Flipchart contained minimal wording, only the health professional side of the Flipchart was used for this analysis. The online tool utilizes five different readability measures (Flesch-Kincaid Grade Level, Gunning Fog Index, Coleman-Liau Index, SMOG Index, and Automated Readability Index). Each readability measure uses a different formula to provide a readability level equivalent to a typical US school grade that would find it easy to read. An average readability school grade level is then calculated from all five measures. We aimed for an average readability score of grade five for the patient resources (meaning any patient who has finished at least grade five in school would find this easy to read), and grade nine for health professionals’ resources (as recommended by the Working Party).

#### 2.2.4. Focus Groups with Health Professionals

Were conducted at each site jointly by a female physician and Tobacco Treatment Specialist (YBZ) and a female Aboriginal research assistant (MB), both currently PhD candidates. MB has previous experience conducting qualitative interviews and focus groups among Aboriginal participants.

In total, three focus groups were conducted, with 7–9 participants in each group, and a total of 24 health professionals, until reaching data saturation, meaning that no new findings or themes were generated. Participants included three GPs, 6 midwives/nurses, 6 AHW; and 9 other allied health workers. Each focus group was approximately one hour in length, and included light refreshments. A semi-structured interview guide was developed across the same six themes used for the expert panel feedback analysis (Attraction, Comprehension, Self-Efficacy, Graphics and Layout, Cultural acceptability, and Persuasion) (Supplementary Materials: Appendix 1). The aim of the focus groups was to receive feedback on the draft version of the resources and suggest changes that would improve them. All of the health professionals treating pregnant women from the service were invited to attend. No information was collected on health professionals that chose not to attend the focus group from these services. Only the participants and researchers were present at the time of the focus group. In the NSW group, the medical director of the service, who also works at the service as a GP, participated.

Focus groups were audio-recorded and professionally transcribed. Transcribed data were coded using Nvivo 11 software. Analysis conducted by one researcher (YBZ) was checked by a second (MB) for the six pre-determined themes. Thematic analysis for emerging themes was conducted by both researchers (YBZ and MB) using a general inductive approach [35]. Coding was discussed until agreement was reached. This enabled researcher triangulation and helped ensure that the meaning of the analysis was the same between the two coders to enhance validity and reliability of the findings, and reduce personal bias.

#### 2.2.5. Ethics

The study was approved by the University of Newcastle Human Research Ethics Committee (HREC) (Reference H-2015-0438); by AH&MRC Ethics Committee (Reference #1140/15); by AHREC Ethics Committee (Reference #04-16-652); and by the Far North Queensland Human Research Ethics Committee (HREC) (Reference #16/QCH/34-1040).

#### 2.2.6. Reimbursement

The medical service/staff performing the SAM scoring received an $80 shopping voucher.

## 3. Results

### 3.1. An Expert Scientific Panel

A detailed summary of all the expert panel feedback is provided in Appendix 2 (Supplementary Materials). Overall, all experts agreed that the attraction and cultural acceptability of the resources were high. Some made specific recommendations on sensitive issues, such as ways to negotiate a smoke-free home with Elders; or suggestions for more acceptable and easily understandable wording for Aboriginal women. Minor suggestions were made about the graphic and layout to make the resources more practical and useful (e.g. highlighting certain information, and adding more visual references). Specific words were suggested to simplify the patient resources and additional information to aid self-efficacy and comprehension including electronic cigarettes; harm reduction; depression; family/household smoking; and women’s perception on the use of NRT in pregnancy. Additional text was suggested to be consistent with a non-judgmental communication style.

### 3.2. The Suitability of Materials (SAM) Assessment Method Score

All of the patient brochures were scored as suitable by the staff members. Two brochures received a mean score above 70%, indicating a superior material (Table 1), and the rest of the brochures were perceived as adequate, with their mean score close to the cut point indicating a superior score. A consistent rating for the NRT brochures under ‘Layout’ was that the material looked “uninviting and discouragingly hard to read”. The interrater reliability was found to be poor with Kappa = −0.75 (*p* < 0.028), 95% CI (−0.939, −0.177).

### 3.3. Readability Testing

The average readability score for the patient resources was 6.4 (range 5.1–7.2), and for the health professionals’ resources, 9.8 (range 8.5–10.6) (Table 1).

### 3.4. Focus Groups with Health Professionals

#### 3.4.1. Pre-Determined Themes

Two sets, each with two pre-determined themes, were closely related to one another with the same two themes coded to the same sentences. Therefore, each set was grouped together as one theme (1. Graphic and Layout impacting Attraction; 2. Self-efficacy and Persuasion), forming four distinct themes:

##### Graphic and Layout impacting Attraction

Overall the health professionals found the resources attractive, especially the pictures used for the flipchart “The pictures are beautiful, absolutely….They’re gorgeous girls… no horror stories there. They’re real” (*SA*).

They suggested the treatment manual was too long and needed to include more visual devices such as graphs, boxes and tables.

*“Reading* *a whole manual like this is not going to happen….There’s too much writing” (NSW); “I like more tables, graphs, pictures, because I don’t have to go double…I don’t like reading pages long. I’ll just look at it and go 'Yeah, too much.'” (QLD).*

The desktop guide was perceived as too large and confusing, and was suggested to be converted to a mouse pad “*our desk is too small (NSW)… Maybe if it was a mouse pad (Qld)”*. The layout of the NRT treatment algorithm was advised to be simplified, so that actions required by the health provider are described in boxes, and patient assessments in arrows between boxes “*It’s not really clear to me how–what the categories are in each box.” (NSW).*

##### Comprehension

Across the three states, health professionals had sound comprehension of the content within the resources, and agreed they were comprehensive *“Content wise it's pretty good” (SA) “The actual information is good” (QLD) “There’s good stuff in here” (NSW).*

##### Self-Efficacy and Persuasion

Health professionals found the resources useful and helpful to engage in the conversation about smoking with the pregnant woman *“…this little chart thing* (referring to a table describing the risks versus benefits of using NRT during pregnancy) *would be really, really good for the doctor to go through” (SA) “…some of my clients, I know what I’m going to address next time I see them, I’ll probably go through this more myself” (NSW).*

They had various suggestions to increase the usefulness of the resources, including aggregating all of the brochures into one booklet “*people will nod very nicely and say “thank you very much” but if you go outside, they’re in the bin…. as a book then she could look at the picture and I could run through this and discuss it with her” (NSW);* and having the videos available for them to show the woman *“I think it’d be more interactive if we had iPad in there also because then you could* (show them the videos*)” (NSW).*

##### Cultural Acceptability

Health professionals found the resources to be appropriate for the communities they treat, especially the use of photographs of Aboriginal women from diverse communities and backgrounds *“Because it's got different sorts of girls on it…” (SA); “see your own representation in the flip chart to relate to. Like 'That could be me” (QLD).*

The Qld focus group remarked on the absence of a Torres Strait Islander photograph *“I don't know if you've got any Torres Strait Islander women in there” (QLD).*

#### 3.4.2. Emergent Themes

Four emergent themes arose from the data: ‘Getting the message right’; ‘Engaging with family’; ‘Needing visual aids’; and ‘Requiring practicality under a tight timeframe’.

##### Getting the Message Right

Health professionals were very cautious about using certain words or phrases. This was conveyed for two reasons: firstly, so not to upset the woman *“…you can't really say that to a smoking mum….(SA) She could turn around and say 'I smoked with my other kids, so you think there's something wrong with them?'”(Qld)*; and secondly, to make sure that the message was getting across *“if you go through things like increases the risk of stillbirth and cognitive impairment and impaired lung development, that’s going to be more of a hitting home than 'small baby'” (SA).*

The NSW focus group focused on “how” to utilize the educational material to guide the conversation. Health professionals wanted resources that they can discuss jointly with the woman, *“I normally go through stuff and, okay, this says most people smoke at different times so what do you think is relevant to you, and you’ve got a picture to look at but you’ve also got the prompts” (NSW).*

##### Needing Visual Aids

Recommendations focused a lot on visual devices that could help both engage the woman in the conversation, but also help “getting the message right”.

*“…with* *the community that we’re looking after, it’s about the visual” (NSW).*

*“I'd like* *these more as like posters around the counselling room even…. Because that would generate a conversation with me about all those things anyway.” (SA).*

Specific suggestions were made for posters that could be hung in consultation rooms. One idea was a poster to explain the different types of NRT products available, and a separate one visually showing the differences between NRT (delivering just nicotine) and smoking a cigarette (delivering thousands of different harmful chemicals in addition to the nicotine).

*“…the* *pictures of people actually using it (NRT), I think that would be really helpful.” (NSW).*

*“I’d have, like, that* *big and then with nicotine and then that big with just nicotine because I like to say that to them… that’s one of the messages I always try and say…” (NSW).*

##### Engaging with Family

The importance of family and community within healthcare for Aboriginal people is an area health professionals were particularly aware of. Smoking among other family members was mentioned as a barrier *“the women are trying to quit but they live with a bloke who’s still smoking in the same house” (NSW)*; *“That's a support (family) that women are often very concerned about when they try and quit smoking” (SA).*

Health professionals wanted the resources to address this more in depth and provide useful information to guide the discussion *“…everybody's family and everybody's support network is very, very different, there could probably be a bit more of a focus on 'Okay, this is in specific how we could help you and how your family members could help you…” (SA).*

The importance of family and community was also requested to be integrated in the photography used in the resources, moving beyond pictures of only women and babies.

*“at least include them* *so that visually you know that there are other people that would be smoking in the home.” (Qld); “why is there not a picture of a father with a child and the baby, the mother and the father and the child?” (NSW).*

##### Requiring Practicality under a Tight Timeframe

When discussing the graphic and layout of the resources, multiple suggestions were made to increase the practicality of the resources. Suggestions included making the resources easy to use and fast to find the exact information you need, i.e., adding tabs, having important key information highlighted in boxes, and offering an online version with hyperlinks in the table of contents.

*“We have so many pieces* *of paper floating around, when you need them, you cannot find them. I need something simple, to the point that’s easily done” (NSW); “I'd probably be want to be able to flip to it really quickly…tabs would probably be better for me” (SA).*

Time was mentioned frequently as a barrier, both from the health professionals’ point of view *“…clinical time is so precious at the moment because of the amount of people you’ve got to access on that particular time …” (NSW),* as well as from the patients’ perspective “*most of our pregnant clients have other kids that they didn’t leave home …their ability to concentrate…is limited … And the partner’s been dragged along and he doesn’t necessarily want to be there for a whole lot of stuff or somebody else has been left in the car …Time is a challenge” (NSW).*

### 3.5. Summary of Changes to the Educational Resources Package

Following the above processes, results were summarized and presented to the SCAAP to discuss and agree on the changes that were required. Each medical service also received a community report to distribute to their community members, health professionals, and board for feedback. A summary of the changes that were made is detailed in Table 1. Readability scores improved (meaning they became more readable–i.e., scores were reduced) for all of the educational resources, both for the health professionals–average readability score of grade 8.1 (range 7.1–8.9), and patient booklet with an average readability score of grade 4.7. Unfortunately, due to time constraints, additional photographs with Torres Strait Islander women and/or family members were not feasible. This updated resource package is included as one of the components of the ICAN QUIT in Pregnancy intervention, which in 2017 was pilot tested in six ACCHS across NSW, SA and QLD [26].

## 4. Discussion

### 4.1. Summary of Main Findings

A multi-level evaluation was conducted with an expert panel, a SAM assessment, readability testing, and focus groups with 24 health professionals in three Australian states. Multiple suggestions were made during this evaluation process to improve the usefulness and acceptability of the educational resource package:

- Additional information was required, such as how to deal with a family member who smoked in the house;
- Simplification of words was recommended to increase readability and comprehension;
- Increasing the practicality to allow faster access to information;
- Adding different visual aids to increase engagement and guide the consultation;
- Suggestions were made on how to improve wording to become more culturally responsive for Aboriginal women;
- Recommendations were made on how to facilitate health provider discussions on NRT use during pregnancy, which is a unique barrier for health professionals providing smoking cessation care during pregnancy.

### 4.2. Comparison with Other Literature

Previous research looking at the readability and suitability of educational resources for various health conditions have found that, in general, many are rated as non-suitable and with too high readability scores [36,37,38,39,40]. Many of these studies utilized readability and/or suitability measures, but without a participatory approach where end-users views on the health education material were assessed. In our study, the focus groups and expert panel provided the largest amount of information and recommendations for change.

A parallel analysis was conducted through focus groups with Aboriginal women on the patient-dedicated resources for this intervention (Bovill et al., unpublished data, 2017). Similar to the health professionals in our study, Aboriginal women were supportive of the cultural acceptability of the resources, suggested one booklet, and wanted *‘more information’* on specific harmful effects of smoking. They also requested that the resources would be *‘more engaging’* including real stories of Aboriginal woman who quit smoking during pregnancy. Women also asked for information on non-NRT options to deal with cravings, illustrating that the use of NRT during pregnancy is a unique barrier for both health professionals and pregnant women. As mentioned previously, a similar process has been successfully used in the past for a culturally targeted smoking cessation program for American Indians [24]. Those pre-tested resources were subsequently used for a multi-component intervention in a randomized controlled study. The intervention showed promising results with self-reported 6 month intention to treat point prevalence abstinence rates significantly higher in the intervention group (20.1% compared to 12.0%, *p* = 0.029) [25].

Other smoking cessation interventions with Indigenous people [41] have described using a participatory approach in designing their intervention and resources [42,43], but only one study reported conducting a pre-test on their resources before rolling out the intervention [44]. This might be a contributing factor as to why these interventions did not show a higher smoking cessation rate compared to non-culturally tailored interventions [41,42]. An association has been found with conducting a pre-test and the reporting of cultural challenges by organisations developing tobacco control messages for Aboriginal Australians [45]. Programs not conducting a pre-test may be less aware of the requirements for cultural sensitivity.

The emergent themes from the health professionals’ focus groups are consistent with previous research on barriers and facilitators to smoking cessation care during pregnancy [6,7]. Lack of time was mentioned as one of the most important barriers in a recent Australian cross-sectional survey of GPs and Obstetricians [8], and has also been mentioned in other surveys globally [6]. Health professionals report facing multiple high-priority issues that they need to address during a consultation, and therefore require the resources to aid them in a timely manner [7]. Smoking rates across Aboriginal communities are high, an average of 39% among adults [46]; therefore, smoking may be considered a norm in these communities [21] and has been shown to be an important barrier to quitting in pregnancy [20,21]. Health professionals require specific recommendations on how to address this topic. Visual devices have been shown to be imperative in Aboriginal communities and previous research has identified this need [47,48,49].

### 4.3. Strengths and Limitations

The major strength of this study was the community-based participatory research approach. The resources were developed collaboratively with a working party from two ACCHS including health professionals and community members, and then received input from numerous health professionals working in ACCHS, including Aboriginal Health Workers from those communities. AH&MRC ethical guidelines recommend community ownership: an important aspiration when working in Aboriginal research. Developing the educational materials collaboratively, and consulting with community members on these materials prior to commencement of the project, are factors that contribute to this ownership. Another strength was the multiple methods used to collect data, aiding in research and data triangulation. Readability was assessed both on objective scales, and with a more subjective evaluation (SAM), and comprehension was also assessed via input from health professionals.

There were several limitations that may have impacted on this study. Only three communities were included, and the results might only be representative of those communities. Despite this, the fact that these communities were diverse and from three different states, with similar results across the communities, suggests that these resources might be acceptable and useful for other ACCHS and communities. Another round of community input after the changes were done was not feasible. This is mitigated by the fact that the SCAAP gave constructive feedback on the revised resources. In 2017, a pilot study with six ACCHS across three states was conducted [26], using these resources as part of the intervention. Further feedback and data are being collected on the usefulness of these resources through surveys and interviews from the pilot participants. Due to logistic reasons, focus groups were held with all types of health professionals together. This raises the possibility of a power differential between doctors, nurses and AHW, which might have impacted the expression of their respective views, leading to an over representation of doctors’ views compared to AHW or nurses. As midwifes and AHW are the main point of contact for a pregnant woman during her ante-natal care, under-representation of their respective views might have meant that not all of the issues were identified. As focus groups included a range of health care providers, we were unable to present the data according to the different types of health professionals. Focus groups were not conducted by an independent party, but by the co-authors of the resources. Furthermore, social desirability bias with the SAM scoring and focus groups cannot be excluded, which might indicate that the resources are less acceptable and useful than perceived in this study. However, in the initial explanation about the study, the facilitators emphasized that the purpose was to receive as much feedback as possible to improve and change these resources and were eager to hear both negative and positive viewpoints.

Scores from the SAM differed greatly for the same material resulting in a low inter-rater reliability measure, and did not contribute much to the decision-making on the changes for the resources: The SAM may be thus more subjective and may require several assessments with different people.

### 4.4. Implication for Policy and Practice

These resources were drafted by a tobacco treatment specialist with years of experience in smoking cessation and training health professionals (YBZ), together with an Aboriginal cultural liaison and researcher (MB); and developed jointly with a working party that included health professionals and community members from two ACCHS. The whole process was overseen by a senior researcher who is also a tobacco treatment specialist and GP, and experienced in development of Aboriginal smoking cessation resources (GG). Despite this, many changes were needed to assure these resources were useful and appropriate. The findings from this study highlight why an evaluation process is important and justified and should be adapted as a requirement when developing educational resources, prior to rolling them out for practice. Despite educational resources being very common as part of behavioural change interventions, many of them lack a formal evaluation process, or this process is not included as part of the intervention description. The process described here is an example of what might be used in future interventions with diverse populations. However there are many other approaches to evaluation [50], such as the Cloze Test that assesses readability and comprehension together [51].

There are multiple educational resources being developed for various health conditions. In fact, most organizations and interventions develop their “own” branded resources. This is time consuming and potentially an uneconomical use of resources. Instead of multiple different resources, national peak organizations and/or the Department of Health should be focusing on developed targeted resources that are evidence based, cultural acceptable, useful, and shared nationally for free. These “template” validated resources could then be used by other organizations, projects and interventions. The process described in this study was time consuming (over a year) and required funding (including travel costs, reimbursement, research assessment time, and transcribing) that might deter other projects from undertaking such a process. This supports having a well-developed regularly updated national “bank” of validated educational resources that can be used freely by anyone. Having a national bank as suggested might still require validated resources to be culturally tailored to the specific communities for which they are intended. The complexity of this, combined with the uncertain evidence regarding the added clinical benefit of tailored educational resources [15], makes this a complex issue that requires further research to better understand what would be the most time and cost-effective approach.

The current process has increased the likelihood that the updated resources would be acceptable, useful and culturally responsive among participants of these three communities. By implication, the resources might be suitable for other similarly located Aboriginal communities (in the same three states) and thus appropriate for the second phase of the ICAN QUIT in Pregnancy intervention [26]. However, for phase three of this intervention (a cluster randomized controlled trial), intended to include 30 communities across additional Australian states and territories, further input and changes may be needed to ensure acceptability, usability and cultural responsiveness across all of these diverse communities.

## 5. Conclusions

A structured 4-step evaluation process informed the development of a resource package to be used as part of a multi-component intervention, aimed at improving how health professionals manage smoking in Aboriginal and Torres Strait Islander pregnant women who smoke. The evaluation process elicited specific suggestions for needed changes and improvements to ensure these resources were acceptable, culturally responsive and useful. Health professionals require simple, practical, visual resources that engage pregnant women in a shared conversation on smoking during pregnancy. The generalizability of these findings might be limited and requires more research.

This novel formative evaluation protocol has never been done previously in Australia. If these resources prove effective, the methodology could be adapted for other Indigenous interventions, and culturally diverse programs. The added value of this time-consuming and costly process is yet to be justified in research, and might impact the potential adaption by other projects.

## Figures and Tables

**Table 1:** Summary of Suitability of Resources (SAM) and Readability scores (before and after changes), and changes that were done to the educational resources package.

Resource	SAM Scores (Mean)	Readability Score-Average Grade Level (Range of Sub-Sections)	Summary of Changes to the Resource Materials	Readability Score after Changes-Average Grade Level
Training manual	Not relevant	10.4 (8–13.4)	Additional information was added as suggested: tabs were added; each section was given a different colour theme and prefaced with a colourful highlighted box summarizing the main points; an electronic version with hyperlinks was also provided	8.9
Flipchart	Not relevant	8.5 (4.7–31.4)	Additional information was added: two pages (from the women’s side) were also transformed into A3 posters graphically illustrating the different NRT products, and the differences between using NRT and smoking a cigarette.	8.5
Desk top guide	Not relevant	10.6	Simplified to a three-step process; converted to a mouse pad.	7.1
Patient brochures:				
‘Quitting in pregnancy’	86, 40 (63)	7.2	All brochures were aggregated into one A5 booklet; additional information was added as suggested to enable a shared discussion; Information regarding family member support was added; specific wording was simplified; layout regarding the different types of NRT products was improved, and pictures of pregnant women using NRT were added; blank ‘quit plans’ for the woman to fill out with the health professionals were added.	4.7 (booklet)
‘Triggers’	43, 95 (69)	6.4		
‘Smoke-free homes’	70, 100 (85)	6.5		
‘NRT patch’	73, 43 (58)	6.1		
‘NRT gum’	57, 93 (75)	6.6		
‘NRT lozenge’	43, 91 (67)	6.3		
‘NRT spray’	85, 50 (67.5)	5.1		
‘NRT inhaler’	40, 86 (63)	7.1		

## Supplementary Materials

The following are available online at www.mdpi.com/1660-4601/14/10/1148/s1, Appendix 1: Interview guide for focus groups—Health Professionals; Appendix 2: Summary of feedback provided by the expert panel.
