# Supplementary material for: Assessing and Validating an Educational Resource Package for Health Professionals to Improve Smoking Cessation Care in Aboriginal and Torres Strait Islander Pregnant Women
Source: Int J Environ Res Public Health. 2017 Sep 29;14(10):1148. doi: 10.3390/ijerph14101148 (PMC5664649; doi:10.3390/ijerph14101148)
Supplement: Supplementary file 1 [file ijerph-14-01148-s001.pdf]

Supplemental file - Appendix 1:

Interview guide for focus groups – Health Professionals

Instructions for interviewers

Briefly introduce yourself and explain about the study.

Include main points outlined in the information sheet provided to participants.

Gain Informed consent.

Distribute each resource separately, for individuals to view before discussing with the group

Please allow all participants 5 minutes to review each resource (more if needed) before starting discussion

Encourage participants to refer to the resource in question throughout the session

Encourage participants to make notes as they go on the resources, make sure they understand you will be collecting these at the end.

Note – there are no right or wrong answers. Try not to prompt the participants. Try not to interrupt the responses.

List of resources to be discussed

1. Desk top Guide
2. Treatment Manual
3. Flipchart

Ask the following questions.

1. **Attraction**
   Let’s look at the cover. Would you want to read this?
   If not – could you tell me why?
   What catches your eye?
2. **Comprehension (understanding the content)**
   Could you tell me in your own words what this is all about?
   Anything else?
   Are there any words that you think might be hard to understand? Which ones?
   Do you have any suggestions for improvement as to the content?
   a. Should anything be added?
   b. Should anything be removed?
3. **Self-efficacy**
   Could you use this for treating pregnant patients?
   If not, what doesn’t make sense? What doesn’t seem useful?
   Would you need any other information in order to help them to quit smoking?
4. **Graphics and Layout**
   What catches your eye? What do the pictures tell you?
   Do the pictures help get the message across?
   What do you think of the colours?
   Do you have any suggestions for improvement as to the graphics?
   a. Should anything be added?
   b. Should anything be removed?
   c. Should any particular graphic be altered?
   Is the print big enough? Too big? Is there too much print/too little/just right
5. **Cultural Acceptability**
   a) Do you think this is OK to show to pregnant Aboriginal or Torres Strait Islander women?
   b) Is there anything in here that would make you uncomfortable?
   c) Do you find anything in this that is offensive to you?
   d) Are there any inappropriate parts (wording or graphics)?
6. **Persuasion**
   Do you think health providers would be willing to use this?
   Overall
   Overall, what is your opinion of this?
   Can you think of ways this could be improved?

Appendix 2: Summary of feedback provided by the expert panel

Resource Type	Summary of feedback<br>(according to the themes – Attraction, Comprehension, Self-Efficacy, Graphics and Layout, Cultural acceptability, and Persuasion)
General	Self-efficacy: Add more on family/household smoke<br>Attraction: High<br>Cultural acceptability: High
Treatment Manual	Self-efficacy:<br>• Add more information regarding harm reduction, e-cig, depression<br>• Add more emphasis on asking women what they think about possibly using NRT and why with efforts being made to counter any strong erroneous views they hold. Add specific suggestions regarding how HP might counter incorrect ideas about NRT.<br>• Add more detail regarding practical use of measures to guide decision<br>• Suggested omitting HSI if doesn't affect treatment decision<br>• Adding intro on purpose<br>• Add additional resources available including YouTube videos.<br>• Addition of information on differences between smokers, and their ability to quit (suggestion for an additional graph);<br>• Additional information regarding breaking the habit (breaking the pairing)<br>Graphics and layout: Specific recommendations on things that need to be made more clear, stand out<br>Comprehension:<br>• Minor editing suggestions<br>• Suggesting reframing the Ask to the Ask-Provide-Ask method<br>Cultural acceptability:<br>• Suggested addition on issues regarding smoke free homes and sensitivity with how to communicate this to elders.<br>• Revise the section on cultural competent care to be more in detail and comprehensive
Flipchart	Attraction: High<br>Graphics and Layout: Minor changes to make more useful and understandable Organizing the pages differently<br>Comprehension: Minor wording suggestions to clarify<br>Self-efficacy:

	• Additional quotes to help clarify meaning of communication style recommended<br>• Additional information on difference between harm from cigarette to harm from nicotine; differences between smokers, and their ability to quit (suggestion for an additional graph); cost of NRT; e-cig and chewing tobacco<br>• Additional material as personal handouts, also laminated
**Desktop Guide**	Persuasion: Conflicting (some said helpful, others would not use)
**Brochures – General**	Attraction: High<br>Comprehension:<br>• Change specific wording to simplify<br>• Check guidelines regarding timing in relation to food/drinks
**Quitting in Pregnancy**	Comprehension: Simplify wording
**Triggers**	Self-efficacy: Add ways specifically on stress management<br>Comprehension: Simplify wording
**Smoke Free Home**	Self-efficacy: Add health benefits for children<br>Comprehension: Simplify wording
**NRT Brochures – General**	
**Patch**	Comprehension: Simplify wording
**Gum**	Comprehension: Simplify wording
**Lozenge**	Comprehension: Simplify wording
**Spray**	Comprehension: Simplify wording
**Inhaler**	Comprehension: Simplify wording
